# BCR-ABL Promotes PTEN Downregulation in Chronic Myeloid Leukemia

**DOI:** 10.1371/journal.pone.0110682

**Published:** 2014-10-24

**Authors:** Cristina Panuzzo, Sabrina Crivellaro, Giovanna Carrà, Angelo Guerrasio, Giuseppe Saglio, Alessandro Morotti

**Affiliations:** Department of Clinical and Biological Sciences, San Luigi Hospital, University of Turin, Orbassano, Turin, Italy; Università degli Studi di Firenze, Italy

## Abstract

Chronic myeloid leukemia (CML) is a myeloproliferative disorder characterized by the t(9;22) translocation coding for the chimeric protein p210 BCR-ABL. The tumor suppressor PTEN plays a critical role in the pathogenesis of CML chronic phase, through non genomic loss of function mechanisms, such as protein down-regulation and impaired nuclear/cytoplasmic shuttling. Here we demonstrate that BCR-ABL promotes PTEN downregulation through a MEK dependent pathway. Furthermore, we describe a novel not recurrent N212D-PTEN point mutation found in the EM2 blast crisis cell line.

## Introduction

The tumor suppressor PTEN is one of the mostly mutated tumor suppressors in cancer [1]. PTEN acts as a phosphatase that dephosphorylates phosphatidylinositol 3,4,5-trisphosphate (PIP3), the lipid product of the class I phosphoinositide 3-kinases (PI3K). PTEN inactivation by point mutations or deletion aberrantly activates PI3K–AKT pathway, promoting cell growth, survival and proliferation. Beside canonical genetic loss of function, PTEN tumor suppressive functions could be impaired by non genomic mechanisms, such as epigenetic silencing of PTEN, post-transcriptional regulation by non-coding RNAs and post-translational modifications [2], [3]. In particular, deregulation of PTEN protein levels by non-coding RNAs and impaired proper nuclear-cytoplasmic shuttling by mono-ubiquitination have extensively been reported to impair its tumor suppressive functions [1], [4]. Chronic Myeloid Leukemia is a myeloproliferative disorder characterized by the translocation t(9;22), coding for the chimeric protein BCR-ABL [5], [6]. PTEN has been shown to play a critical role in the pathogenesis of Chronic Myeloid Leukemia (CML), through both protein down-regulation and impaired nuclear/cytoplasmic shuttling by mono-ubiquitination [7], [8]. Expression of BCR-ABL was associated with the reduction of PTEN levels although the mechanism of PTEN expression regulation was not reported [9], [10]. The relevance of the BCR-ABL-induced PTEN downregulation and delocalization could have dramatic implications from the therapeutic standpoint. The identification of the mechanisms that promote BCR-ABL-induced PTEN downregulation and delocalization could indeed be targetable and therefore leading to the reactivation of the tumor suppressor with dramatic consequences.

## Results and Discussion

**Figure 1:**
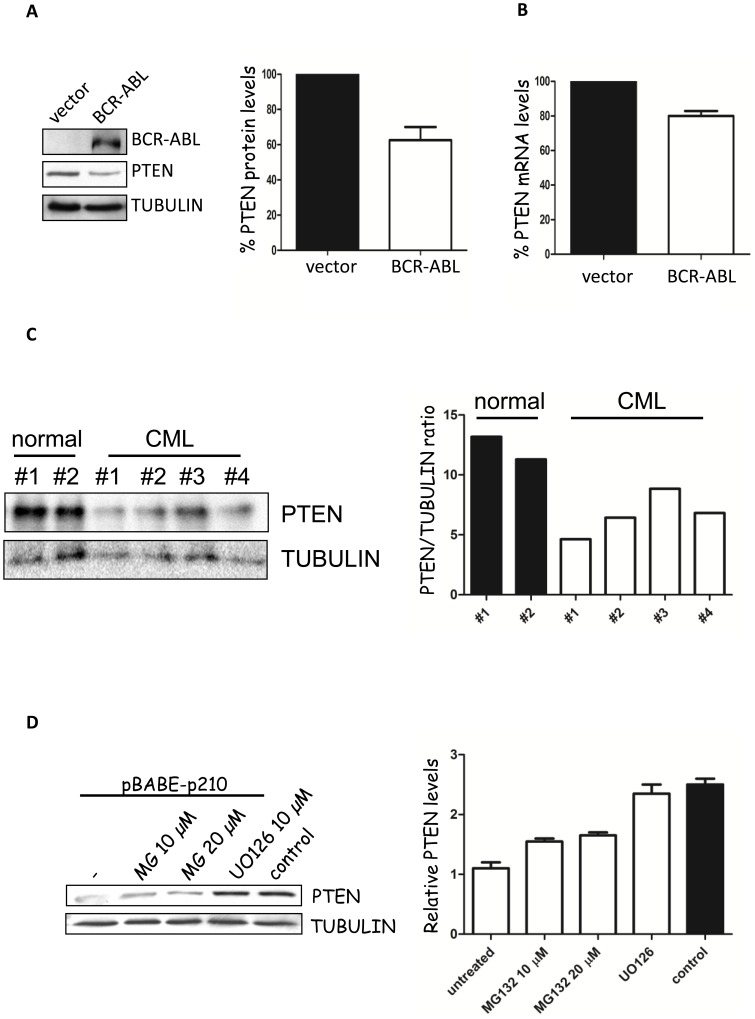
BCR-ABL downregulates PTEN expression. A) Left panel: evaluation of PTEN levels of expression in BCR-ABL-pcDNA3.1 transfected NIH3T3. Right panel: quantification of PTEN levels. B) PTEN mRNA levels in BCR-ABL transfected NIH3T3 cells. C) Left panel: PTEN levels by western immunoblot in primary CD34 positive cells obtained from the bone marrow of normal individuals and Chronic Myeloid Leukemia patients. Right panel: quantification of PTEN levels. D) Stably expressing pBabe-BCR-ABL infected NIH3T3 cells were treated with MG132 and UO126 for 8 hours. Control: parental NIH3T3 expressing pBABE-empty vector.

To better investigate the regulation of PTEN expression levels by BCR-ABL, we have transfected BCR-ABL in NIH3T3 cells and we have measured PTEN levels by western immunoblot. As observed in Figure 1A, similarly to what described elsewhere [9], [10], BCR-ABL expression is associated with the downregulation of PTEN expression, which appears to be transcriptionally mediated (Figure 1B). Similar observations were obtained in the BCR-ABL expressing 32D cell line (data not shown and [9]). CML primary CD34 positive cells also expressed lower levels of PTEN compared to normal bone marrow CD34 positive cells (Figure 1C).

The observation that BCR-ABL downregulates PTEN levels suggested that BCR-ABL could control those pathways that have already be linked to the regulation of PTEN protein expression. Oncogenic Ras-val12 promotes PTEN downregulation through a MEK-Jun pathway [11]. Due to the relevance of Ras-MEK pathway in the signaling transduction by BCR-ABL, we tested whether treatment with MEK inhibitor UO126 could rescue PTEN levels of expression. As shown in Figure 1D, UO126 restores normal PTEN levels in BCR-ABL expressing cells. Interestingly, proteosome inhibitor MG132 is also associated with a slight increase of PTEN levels. The observation that MEK inhibitors upregulate PTEN levels could contribute to explain the original observation that MEK inhibitors in association with TKI are effective in promoting CML stem cell apoptosis [12].

**Figure 2:**
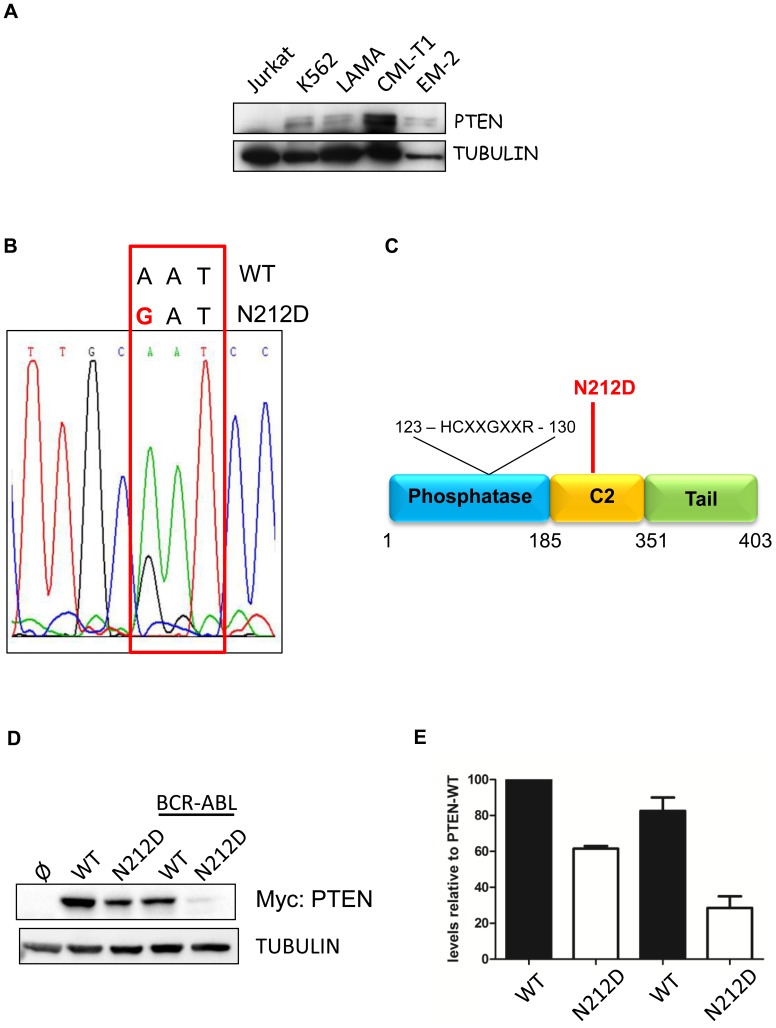
PTEN in CML blast crisis. A) PTEN protein levels in CML blast crisis. Jurkat cell line was used as a PTEN null cell line. B) Chromatogram from sanger sequencing analysis of PTEN in the EM2 cell line. C) Schematic representation of PTEN. D) Left: evaluation by western immunoblot of myc-tagged-PTEN-N212D point mutation expression after transfection of myc-tag-PTEN-WT and N212D plasmid in 292HEK cell line. Right: quantification of PTEN levels.

All together these data further attribute to PTEN the role of tumor suppressor in human CML. Due to the nature of CML as a multiphase disease that can eventually evolve into a blast crisis, we speculate that PTEN could be deleted or mutated during the progression of the disease. It was already shown that in 5 cases of chronic phase CML and in 5 cases of CML blast phases PTEN maintained the wild-type status [13]. We additionally analyzed 5 chronic phase and two blast phases CML patients but we did not observed any PTEN mutations. To further extend this analysis, we assessed the expression of PTEN in four CML blast crisis cell lines (K562, LAMA, CML-T1 and EM2) by western immunoblot. All these cell lines expressed PTEN although at different levels (Figure 2A).

Next, we assessed PTEN mutational status in the four CML blast crisis cell lines, by sequencing all the exons as reported elsewhere [14]. With this approach, we identified a PTEN N212D point mutation in the EM2 blast crisis, as reported in Figure 2B. PTEN N212D is located inside the C2 domain of PTEN, which is involved in the binding of phospholipid and in the regulation of PTEN protein stability (Figure 2C) [15]. To assess the effects of N212D mutation on PTEN stability, we generated PTEN N212D mutant in pRK5-myc-tagged-PTEN vector by site direct mutagenesis, as reported previously [8]. Expression of PTEN N212D mutant construct in 293HEK cells revealed that this mutation renders PTEN unstable with a consequent marked reduction of its expression (Figure 2D and 2E). In conclusion, we propose that PTEN is mostly involved in the pathogenesis of the chronic phase of CML through non genomic loss of function. In particular, BCR-ABL itself is able to promote both PTEN downregulation and delocalization with the loss of its tumor suppressive function [7], [8]. Progression of CML into blast phase does not seem to be associated with PTEN point mutations that genetically cause complete PTEN inactivation. Additional analyses using highly sensitive next generation sequencing approaches are mandatory to assess PTEN mutational status during the CML progression into blast phase in bigger cohorts of patients.

## Materials and Methods

### Cell line and reagents

NIH3T3, 293HEK, Jurkat, K562, CML-T1, LAMA and EM2 cell line were purchased from ATCC. Myc-tagged pRK5-PTEN, p210-BCR-ABL pcDNA3.1 and p210-BCR-ABL-pBABE plasmids, myc-tag antibody, anti Tubulin antibody and mutagenesis strategy were described elsewhere [8]. Similarly, transfections and infections were performed as previously described [8].

### Western immunoblot

Cells were lysate and treated as described elsewhere [8].

### PTEN sequences

PTEN sequencing analysis has been performed as described elsewhere [14].

### CML primary cells

CML primary cells were collected at the moment of diagnosis from 4 patients upon informed consent. Cells were separated to obtain Lin^−^CD34^+^CD38^+^ and Lin^+^ cells as previously reported [8]. This project was reviewed and approved by the San Luigi Hospital Institutional Ethical Committee (Code # 10/2013).
